# Detecting microsatellite instability by length comparison of microsatellites in the 3′ untranslated region with RNA-seq

**DOI:** 10.1093/bib/bbae423

**Published:** 2024-08-29

**Authors:** Jin-Wook Choi, Jin-Ok Lee, Sejoon Lee

**Affiliations:** Department of Health Science and Technology, Seoul National University, 1 Gwanak-ro, Gwanak-gu, 08826 Seoul, Republic of Korea; Department of Health Science and Technology, Seoul National University, 1 Gwanak-ro, Gwanak-gu, 08826 Seoul, Republic of Korea; Department of Health Science and Technology, Seoul National University, 1 Gwanak-ro, Gwanak-gu, 08826 Seoul, Republic of Korea; Department of Pathology and Translational Medicine, Seoul National University Bundang Hospital, Seoul National University College of Medicine, 82 Gumi-ro 173beon-gil, Bundang-gu, 13620 Seongnam, Republic of Korea; Precision Medicine Center, Seoul National University Bundang Hospital, 82 Gumi-ro, Bundang-gu, 13620 Seongnam, Republic of Korea; Department of Genomic Medicine, Seoul National University Bundang Hospital, 82 Gumi-ro, Bundang-gu, 13620 Seongnam, Republic of Korea

**Keywords:** microsatellite instability, RNA-seq, novel MSI detection, comparing microsatellite length

## Abstract

Microsatellite instability (MSI), a phenomenon caused by deoxyribonucleic acid (DNA) mismatch repair system deficiencies, is an important biomarker in cancer research and clinical diagnostics. MSI detection often involves next-generation sequencing data, with many studies focusing on DNA. Here, we introduce a novel approach by measuring microsatellite lengths directly from ribonucleic acid sequencing (RNA-seq) data and comparing its distribution to detect MSI. Our findings reveal distinct instability patterns between MSI-high (MSI-H) and microsatellite stable samples, indicating the efficacy of RNA–based MSI detection. Additionally, microsatellites in the 3′-untranslated regions showed the greatest predictive value for MSI detection. Notably, this efficacy extends to detecting MSI-H samples even in tumors not commonly associated with MSI. Our approach highlights the utility of RNA-seq data in MSI detection, facilitating more precise diagnostics through the integration of various biological data.

## Introduction

Microsatellites, which consist of one to six base pair motifs repeated throughout the human genome, are susceptible to alteration when the mismatch repair (MMR) system is impaired, leading to the occurrence of microsatellite instability (MSI) [1–3]. Recent studies have underscored MSI’s role as a biomarker in cancers like colorectal, endometrial, and gastric adenocarcinomas, demonstrating its significant implications in research and clinical settings, where MSI-high (MSI-H) colorectal tumors correlate with improved prognosis due to better responses to immune therapies [4–7]. The recent approval of pembrolizumab, a PD-1 inhibitor, for the treatment of MSI-H and MMR-deficient tumors serves as a prime illustration of its clinical significance [8]. Consequently, the effectiveness of MSI as a biomarker in guiding immune checkpoint blockade therapies not only underscores its academic significance but also solidifies its practical relevance in clinical settings.

Traditional assays to detect MSI are MSI-polymerase chain reaction (PCR) of specific microsatellite sites, known as the Bethesda panel, and immunohistochemistry (IHC) to assess the expression of MMR proteins [9, 10]. The advent of next-generation sequencing technologies has revolutionized MSI detection with computational methods like mSINGS, MSISensor, and MANTIS assessing a wider range of microsatellite loci, enabling more detailed analysis applicable to a broader spectrum of cancer types [11–15]. These tools perform MSI detection by comparing the distribution of repeat length of microsatellites, utilizing whole genome sequencing, whole exome sequencing (WES), and targeted sequencing [16]. While standard deoxyribonucleic acid (DNA)–based MSI detection methods are effective, integrating ribonucleic acid (RNA) analysis can provide additional layers of genetic variations, further enhancing the accuracy and scope of the results. Previous studies utilizing RNA sequencing (RNA-seq) for MSI detection, such as those by Li, Danaher, and Pacinkova, have primarily focused on analyzing expression levels of genes to identify gene signatures for predicting MSI status [17–19]. These genes were mostly non-overlapped, and their gene ontology (GO) analysis results showed different repertoires of GO terms [20]. In addition, the area under the receiver operating characteristic (ROC) curve (AUC) value (0.69) observed from expression profiles of some genes in the analysis of endometrial cancer showed limited effectiveness. This indicates that detecting MSI by expression profile potentially lacks consistent robustness in results. It could imply inaccuracies in MSI detection, particularly leading to mistakes in pan-cancer. There is a need for a more accurate and direct MSI detection method in RNA-seq that involves a comprehensive investigation across the microsatellite dataset.

In this study, we have developed a novel tool named Microsatellite Instability Detection with RNA-seq Analyzing Comparison of Length Extensively (MIRACLE), the python package specifically designed to detect MSI status by comparing microsatellite lengths in RNA-seq. We applied this tool to analyze RNA-seq samples from The Cancer Genome Atlas (TCGA), specifically focusing on MSI-prone tumors. These analyses revealed a distinct pattern between MSI-H and microsatellite stable (MSS) cases, and we constructed a machine-learning model to classify the two cases with this pattern. Lastly, we further applied this model to non-MSI–prone tumors to expand its potential applicability in a broader usage. Our research provides another approach to MSI detection, offering enhanced accuracy and practical utility in clinical settings.

## Materials and methods

### Data sets

RNA-seq data of tumor and normal samples from TCGA project were downloaded from GDC portal (https://portal.gdc.cancer.gov/). All of the files were downloaded in bam format that was aligned with human reference genome 38 (hg38) using STAR aligner. We used the Qualimap 2 tool to check the read quality of the data used in our analysis [21] (Supplementary Table 1). MSI status (MSI-H and MSS) of four cancers (colon adenocarcinoma, rectal adenocarcinoma, uterine corpus endometrial carcinoma, and stomach adenocarcinoma) was evaluated by Biospecimen Core Resource at Nationwide Children’s Hospital using a panel of four mono-nucleotide repeats (BAT25, BAT26, BAY40, and TGFBRII) and three dinucleotide repeats (D2S123, D5S346, and D17S250) [22–24]. According to the findings from prior research showing negligible significance in differentiating MSI events between MSI-low (MSI-L) and MSS, our study has chosen to focus solely on MSI-H and MSS for a clearer demonstration of differences [25]. The detailed list of samples and MSI status are given in Supplementary Table 2.

### Defining a total pool of microsatellite repeats

To investigate the length variation in microsatellite loci, we used an exome-wide reference set of microsatellite loci that were originally introduced by previous study [26]. This study identified 386,396 microsatellite loci, using the Sputnik algorithm in the messenger RNA (mRNA) sequences of 39,496 RefSeq genes. As we analyzed different versions of genome from the study, we used the UCSC LiftOver tool with a default option to convert microsatellite bed files from hg19 to hg38. Of 386,396 microsatellite site loci, we discarded unlifted 150 loci, and the final reference set of microsatellite loci in our study comprised a total of 386,246 loci. These microsatellite repeats consisted of 112,865 mono-, 63,143 di-, 132,046 tri-, and 78,192 tetranucleotides. Additionally, these included 154,529 codings, 181,160 3′ untranslated region (UTR), and 50,557 5′ UTR microsatellite loci.

### Calculating microsatellite length

We utilized the Pysam package (v. 0.15.3), a tool in Python for reading, manipulating, and analyzing bam files [27]. We extracted sequence reads that overlapped with the microsatellite loci of total reference pool. The sequence of microsatellites within overlapped reads were compared with the reference microsatellite repeats. To discount truncated microsatellite repeats, we considered only microsatellite where the 2 bp flanking sequences were identical with matching reference repeats (both 5′ and 3′). We collected the array of lengths of microsatellites in each locus, ensuring a minimum length of five, and generated the length profile in each sample. We designated this profile as a “Length list”.

### Making reference normal with invariable microsatellite

To construct a reference length profile that represents the characteristics of the available normal sample, we extensively examined the “Length list” of normal samples in each cancer type. For each microsatellite locus within normal samples, we measured both the modal length and the frequency of the occurrence of the microsatellite at this modal length. We designated the microsatellite loci as “Invariable microsatellite loci”, where over 95% of the normal samples shared the same modal length, and calculated the median value of frequency at invariable microsatellite loci. Subsequently, we utilized the modal length and its median value to generate a “Reference normal” by repeating the modal length for each locus a number of times equal to the median value.

### Detection of unstable microsatellite

We assessed the difference of the length profile between tumor samples and matched or reference normal samples, using the Kolmogorov–Smirnov (KS) test for statistical evaluation. Minimum of five sequence reads in each microsatellite locus were considered for evaluation. We established a false discovery rate (FDR) threshold of less than 0.05 to determine statistical significance. The loci satisfying these conditions were designated as “Unstable microsatellite loci”. In the quantitative analysis of unstable loci within MSS and MSI-H groups, we utilized the ggplot2 package in R for data visualization [28]. To statistically assess the differences in the number of unstable loci between these two groups, we used the Wilcoxon rank-sum test (α = 0.05).

### Microsatellite instability status prediction

We developed binary classifiers for predicting the MSI status of four cancer types (colon adenocarcinoma, rectal adenocarcinoma, uterine corpus endometrial carcinoma, and stomach adenocarcinoma) using random forest models implemented through the Python scikit-learn package (version 0.21.3, n_estimators = 100) and XGBoost models [29–31]. Every sample was encoded with the number of unstable microsatellites. To ensure balanced sampling between MSI-H and MSS samples, we divided the samples into two groups and conducted random sampling within each group. We selected 80% of the MSI-H group samples and an equal number from the MSS group to obtain the training data and performed 10-time cross-validation. To assess the precision of our models for MSI status prediction, we calculated the AUC value for each type of cancer.

Subsequently, we extended the application of our developed models to test their efficacy in predicting MSI status across the non MSI-prone tumors. Given the absence of predefined MSI status for these additional cancer types, we followed the previous research that conducted MSI status prediction on these cancer types [26]. Consistent with our methodology for MSI-prone tumors, we measured the number of unstable microsatellites in non MSI-prone tumors in the same manner. We then utilized our classifying models to calculate the AUC for each cancer type. The value of probability is calculated for each sample, the fraction of trees in the forest voting for MSI-H sample.

**Figure 1:**
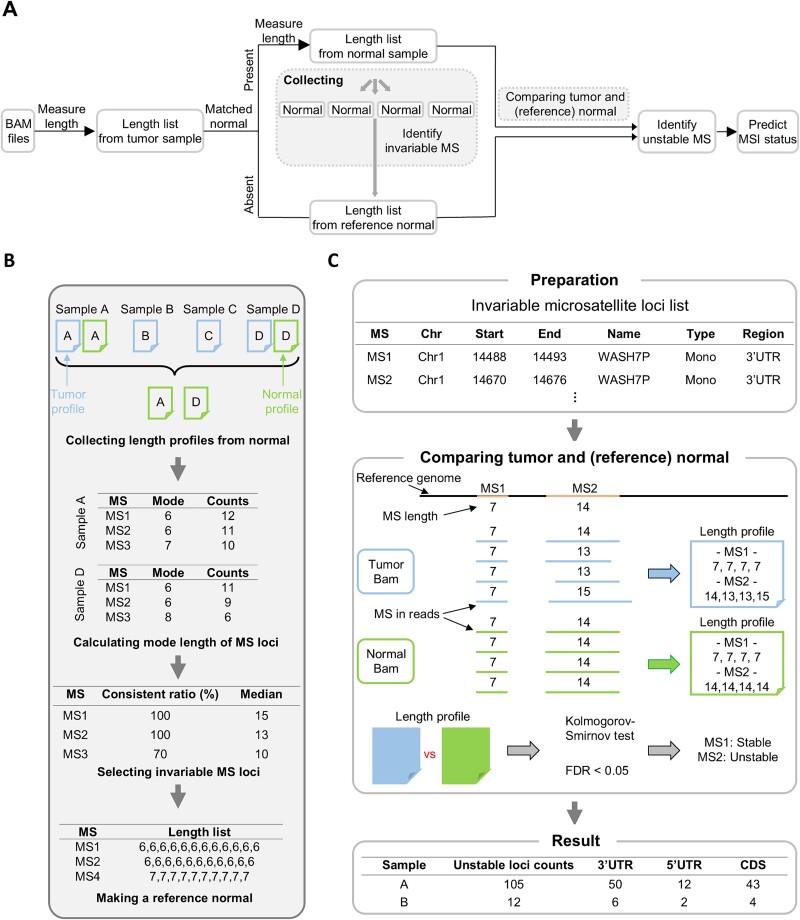
Schematic illustration of detecting MSI by comparing length of microsatellite in RNA-seq. (a) Overview of the analyses pipeline. Microsatellite length was measured for each sample from tumor BAM files. Microsatellite length was also measured from matched normal files if available, or reference normal lengths were used otherwise. Compared microsatellite length distributions to detect unstable microsatellites and predicted MSI status. (b) Detailed process for generating reference normal. (c) Statistical analysis for comparing the length profiles. The KS test assessed differences of microsatellite length for significance, with an FDR below 0.05. MS denotes for microsatellite.

### Performance comparison

To evaluate the performance of MIRACLE in four MSI-prone tumors, we compared it with MSISensor-RNA [32]. We used the informative gene list provided in the MSISensor-RNA study for MSI popular cancers. The expression values for these genes were downloaded from the GDC portal (https://portal.gdc.cancer.gov/). As recommended, we used the log_2_(1 + FPKM) values for the expression data. Using the train command, we created a custom model with same training data sets used in MIRACLE, and then evaluated its performance using the detection command.

## Results

### Overall scheme of the study

In this study, we introduced MIRACLE, a python package to investigate the microsatellite length variation in RNA-seq data (Fig. 1a). Our investigation utilized RNA-seq bam files from TCGA database (accessible at https://portal.gdc.cancer.gov/). The investigation encompassed 1,352 samples from 4 MSI-prone cancers: colon adenocarcinoma (COAD), rectal adenocarcinoma (READ), uterine corpus endometrial carcinoma (UCEC), and stomach adenocarcinoma (STAD; Table 1). We examined 386,246 microsatellite loci in total, following the update of microsatellite repeats in 39,496 RefSeq mRNA sequences to the hg38 (GRCh 38) version. Microsatellite lengths within sequencing reads were measured at each locus, generating detailed length profiles for these loci in every sample (Materials and methods). By comparing the microsatellite lengths obtained from each sample, we measured DNA slippage events, enabling the creation of a model that predicts MSI status.

**Table 1:** Number of RNA-seq samples in four cancer types.

Cancer type	The number of RNA-seq	The number of MSI-H sample	The number of MSS sample
COAD	361	76	285
READ	147	4	143
UCEC	490	170	320
STAD	354	79	275
Total	1,352	329	1,023

### Filtration of microsatellites for accurate microsatellite instability detection in ribonucleic acid-sequencing analysis

Of 386,246 loci, we focused on loci that were not influenced in microsatellite length by expression levels (Fig. 1b, Supplementary Fig. 1). We analyzed normal samples in each cancer to select loci with invariable lengths that exhibited consistent length profiles across most samples, which we termed “invariable microsatellites” (Materials and methods). Through statistical analyses, we compared the length distribution of invariable microsatellites in both tumor and matched normal samples (Fig. 1c, Materials and methods). We discovered loci with significant differences, which indicated frequent MSI events occurrence at these sites, leading to length variation. This condition reflects instability, and we designated these loci as “unstable microsatellites”. The quantification of unstable microsatellites enabled us to assess the degree of MSI event present in each sample. This measure was subsequently adopted as a quantitative indicator for evaluating MSI status.

### Development of cancer-specific reference normals for enhanced microsatellite instability detection in ribonucleic acid-sequencing analysis

Among the 1,352 RNA-seq samples analyzed, the majority of cases (94%) did not possess matched normal samples. We generated a reference normal in each cancer to facilitate the comparison of microsatellite lengths in these cases (Fig. 1b, Materials and methods). The reference normal was constructed with the available normal samples, designed to represent the typical length features of normal samples. Specifically, the data of reference normal consisted of the most common length and occurrences at each microsatellite loci across the normal samples. By comparing tumor samples with the reference normals, we measured the extent of MSI events of each sample.

### Characterization of invariable microsatellites and microsatellite instability event distribution across genomic regions in four microsatellite instability-prone tumor types

**Figure 2:**
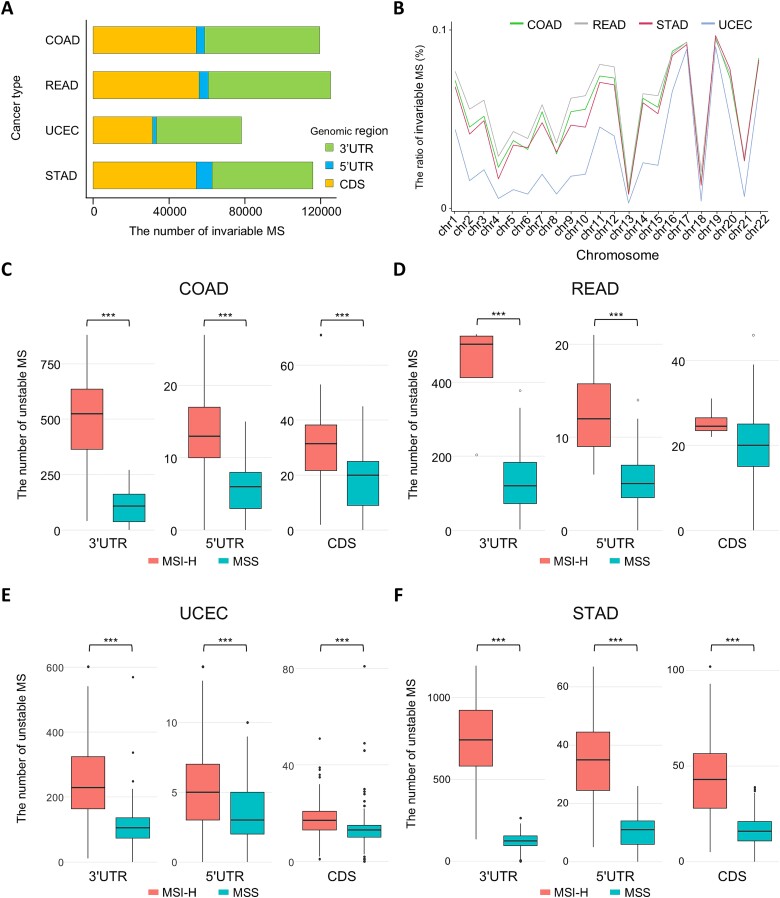
Distribution of MSI event between MSI-H and MSS samples. (a) The number of invariable microsatellite in COAD, READ, UCEC, and STAD is illustrated in the bar chart. The color of bar indicates genomic location of invariable microsatellite. (b) The line chart shows the proportion of invariable microsatellites within chromosomes. The percentages of invariable microsatellites among all microsatellites are displayed in y axis. (c–f) Distribution of the number of unstable microsatellite in four cancers. The boundaries of the box represent the interquartile range; the lines at the center denote medians; and the whiskers extend to values that are within 1.5 times the interquartile range from the median. Color of box shows two types of MSI status: MSI-H and MSS.

Our research first explored the invariable microsatellite in four cancers to understand the comprehensive landscape of MSI. This exploration revealed counts of invariable microsatellites, with 119,786 identified in COAD, 125,166 in READ, 78,306 in UCEC, and 116,002 in STAD (Fig. 2a). Despite the variance in the total counts, the distribution of invariable microsatellites across different genomic regions within these cancer types exhibited a similar proportion. Most invariable microsatellites were located in the 3′ UTR and coding sequence (CDS) regions, accounting for 51% and 45%, respectively. Additionally, the majority of invariable microsatellites consisted of mononucleotide and trinucleotide (Supplementary Fig. 2a and b). Furthermore, our investigation into the chromosomal prevalence of invariable microsatellites showed a notable concentration on chromosomes 17 and 19 (Fig. 2b). The features of these invariable microsatellites align with those observed in previous studies of microsatellites [26, 33]. This consistency suggests that our research has successfully achieved an unbiased selection of invariable microsatellites from the entire microsatellite pool.

Next, to assess the occurrences of MSI events, we quantified unstable microsatellites within MSI-H and MSS samples of the four cancers (Fig. 2c–f). This quantification was conducted across different genomic regions in each cancer, offering a detailed analysis of MSI event distribution. Our statistical analysis showed that the majority of cases (92%) exhibited significant differences in the number of unstable microsatellites between MSI-H and MSS samples. Notably, the most pronounced differences were observed in the 3′ UTR. This finding indicates a high frequency of MSI events in microsatellites located in the 3′ UTR regions in four cancer types. Interestingly, this phenomenon was also consistently observed in DNA data, providing additional validation for our findings [25, 26, 34].

### Machine learning-based prediction of microsatellite instability status in microsatellite instability-prone cancers using consistent microsatellite loci of ribonucleic acid-sequencing

**Figure 3:**
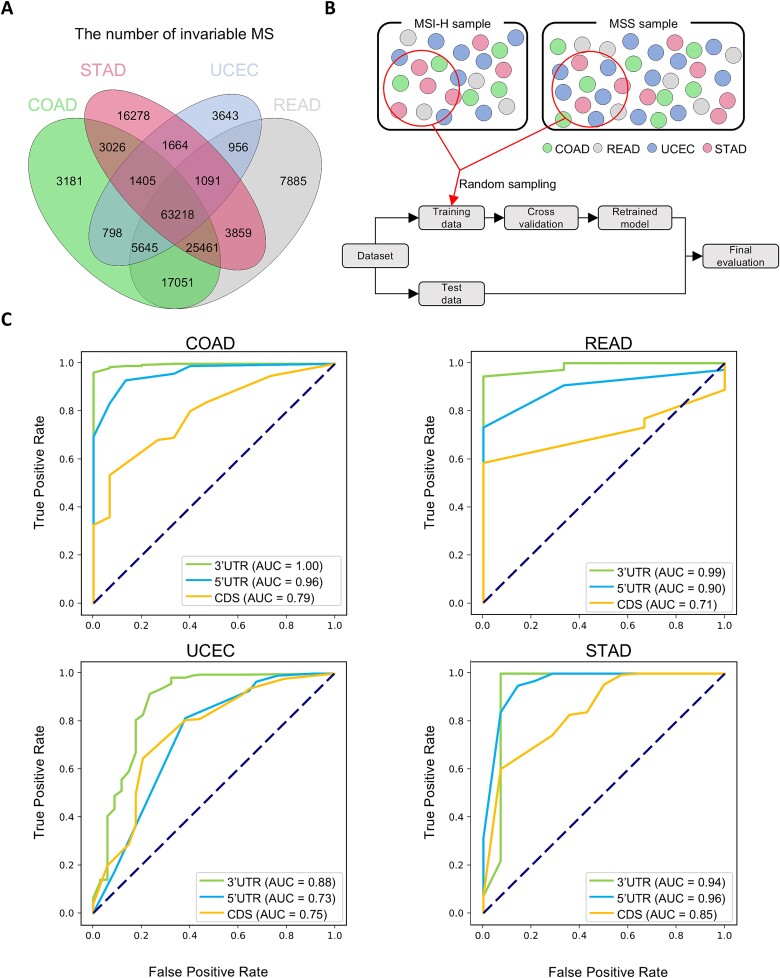
Performance of MSI prediction model in MSI-prone cancer type. (a) Venn-diagram indicates the overlap of number of invariable loci across four MSI-prone cancer types. Each circle represents a unique cancer type with the overlapping areas indicating microsatellites that are consistently invariable across multiple cancers. (b) Flowchart for developing the prediction model. The upper panel delineates how to select training set for the model, and the bottom panel shows the steps for developing the model. (c) AUC scores for model performance in MSI-prone cancers.

Employing machine learning techniques, our study utilized a random forest classification model to predict MSI status in four MSI-prone cancers. To enhance the precision of the model, we focused on the loci where the length of microsatellites appeared to be consistent in four cancer types (invariable microsatellites). We intersected invariable microsatellites of four cancers, finding that 63,218 (41%) consistently retained their length across these cancers (Fig. 3a). Of these microsatellites, we found that 54% were located in the 3′ UTR, 2% in the 5′ UTR, and 44% in the CDS regions. In addition, we verified whether the invariable microsatellites maintained their consistent lengths at the DNA level to check for the effects of inherent RNA-seq noise (Supplementary Table 3). We selected 19 MSI-H cases which had matched normal data available from RNA-seq and compared these normal data with their corresponding WES normal data. After filtering out loci that were not mapped in the WES data, our analysis confirmed that the majority of loci did not exhibit any length variations.

By detecting the number of unstable loci within intersected microsatellites, we developed separate models based on these genomic regions to evaluate how microsatellite location affects the predictive accuracy of MSI status. Subsequently, to construct our training set, we randomly selected 263 MSI-H samples (80%) and an equal number of MSS samples and used the remaining samples to form a test set for model evaluation (Fig. 3b, Materials and methods). We compared the performance of two machine learning models, random forest and XGBoost, and found that XGBoost demonstrated superior performance (Supplementary Table 4). Our ROC curve results, applied to four cancer types, displayed AUC values that fluctuated based on the genomic region (Fig. 3c). Interestingly, the model trained on microsatellites in the 3′ UTR demonstrated high AUC values. This indicates that the model built with microsatellites from the 3′ UTR exhibited the highest performance, aligning with our prior results.

To further evaluate the performance of our model, we conducted a comparative analysis with other MSI detection tools. Traditional MSI detection tools face challenges in utilizing RNA-seq data, such as the requirement for matched normal samples, which are often unavailable in RNA-seq datasets. Consequently, we selected MSISensor-RNA, the most recent and effective expression-based MSI detection tool, for our comparison [32]. We evaluated MSISensor-RNA using the same training and test datasets used for our model. Our analyses indicated that MIRACLE consistently outperformed MSISensor-RNA across all examined cancer types, with higher AUC values, particularly in the 3′ UTR region (Supplementary Table 5).

### Expanding microsatellite instability status prediction to non-microsatellite instability-prone cancer types

**Figure 4:**
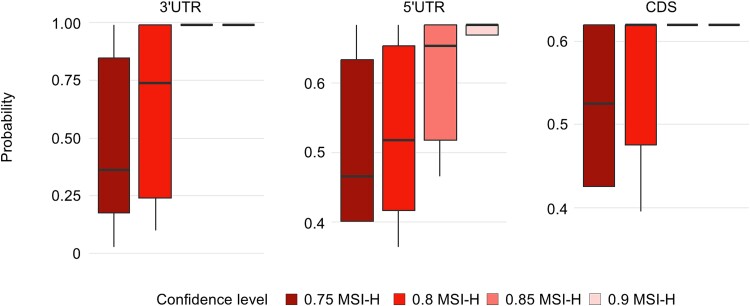
Distribution of probability assigned by the prediction model for samples predicted as MSI-H. The box’s limits depict the interquartile range, while the midlines represent the medians. The whiskers extend to encompass values within 1.5 times the interquartile range from the median. The color of the box indicates the confidence level of the predicted MSI-H sample.

To uncover the expansive potential of our machine learning model, we conducted an extended analysis of 5,595 RNA-seq samples from an array of 15 non MSI-prone cancer types (Supplementary Fig. 3a, Supplementary Table 6). We applied the MSI status information based on the confidence level, where a higher confidence level signifies a greater certainty of the sample being MSI-H in a previous MSI prediction study [26]. At a 0.75 confidence level, 79 samples were predicted as MSI-H, decreasing to 23 at a 0.8 level, 5 at 0.85, and 3 were predicted as above a 0.9 level (Supplementary Fig. 3b). We then evaluated the probability with which our model identified the predicted MSI-H samples as MSI-H, analyzing this throughout differing levels of confidence (Fig. 4). The results showed that as the confidence level increased, our model also predicted MSI-H samples with a higher probability. This suggests that our model accurately detects MSI-H samples even in non MSI-prone tumors. Consistent with our earlier findings, the model built using microsatellites located in the 3′ UTR exhibited the highest performance.

## Discussion

In this study, we introduce a novel method for detecting MSI by measuring the length variation of microsatellites in RNA-seq. However, MSI detection via this method presents two challenges: (i) variability in microsatellite lengths due to individual–specific gene expression levels, and (ii) the scarcity of RNA-seq samples with matched normal. Unlike DNA data, variations in microsatellite lengths in RNA-seq can arise not only from MSI event but also from individual differences in expression levels. To accurately investigate the distribution of microsatellite length, we implemented a filtration process for microsatellite loci. We focused on the loci where microsatellite lengths remain consistent throughout the normal samples (invariable microsatellite), thereby enhancing the accuracy and reliability of our findings. Furthermore, the limitation in comparing length distribution between tumor and normal samples with RNA-seq lies in the frequent absence of matched normal samples. To overcome this challenge, we generated an alternative reference normal for RNA-seq in the absence of matched normal samples. With these resolutions, our observations indicate a distinct difference in microsatellite length variability between MSI-H and MSS samples. These results propose the potential usage of a novel method in distinguishing the MSI-H and MSS samples.

Our model’s performance varies across different cancer types, a phenomenon also observed in other MSI detection tools [13]. We discovered that certain loci are uniquely unstable in specific cancer types, which likely contributes to the observed performance differences [25, 26] (Supplementary Fig. 4). Notably, our findings highlight that the model based on the 3′ UTR microsatellites exhibits superior performance. Several prior studies have reported that MSI events frequently occur in the 3′ UTR rather than the CDS region [25, 26, 35]. These studies suggest that the prevalence of MSI events in the coding regions disrupt protein function, resulting in deleterious effects on the organism [36–39]. To avoid these effect, negative selection occurs, or the disrupted mRNAs are degraded by RNA surveillance mechanisms [40]. Moreover, the 3′ UTR is known to play a crucial role in gene expression, and it has been observed that MSI events occurring in the 3′ UTRs of cancer cells can enhance the stability of oncogene transcripts [41–43]. Therefore, it has been suggested that MSI events in the 3′ UTR may be under positive selection in MSI-H tumors. Our study showed a dramatic occurrence of MSI events in 3′ UTR in MSI-H tumor. The distinction becomes markedly evident in the performance disparity between models constructed using UTR regions and those constructed using CDS regions. Measuring microsatellite length alterations through RNA-seq data has significantly contributed to highlighting this result. Given its cost-effectiveness and wide applicability, this RNA-seq-based methodology is anticipated to provide additional evidence for predicting MSI status in diverse clinics.

We applied our model to non MSI-prone tumors and identified it as effective in accurately predicting MSI-H samples. For MSS samples, evaluation of the model seemed to be difficult because of the absence of a gold standard and an accurate criterion in non MSI-prone tumors (Supplementary Fig. 3a). Detecting MSI in non MSI-prone tumors has been challenging; this difficulty largely arises from tissue specificities and tumor heterogeneity [44–47]. These challenges in pan-cancer MSI assessment could lead to inaccurate MSI status and diagnoses [15, 26, 35, 48]. To address this issue, a multi-faceted approach to MSI detection is necessary [49–51].

In addition, we compared the distribution of MSI events at both the DNA and RNA levels. Interestingly, our findings revealed differences in the distributions of MSI events (Supplementary Fig. 5). We analyzed unstable loci of 19 MSI-H cases where both tumor and normal samples are present in WES and RNA-seq to examine these differences quantitatively (Supplementary Table 7). WES covers only 23% on average for UTR loci, so we focused on unstable loci located in the CDS region for accurate comparison. To accurately identify the extent to which the unstable loci observed differentially in WES and RNA-seq, we considered the loci from two perspectives: (i) loci with read counts under five, and (ii) loci that were significant in the KS test but not in the FDR test. First, we examined each unstable loci in WES and RNA-seq to see if they had fewer than five reads in the other platform data, because loci with fewer than five reads were excluded from the analysis. Low read mapping in these regions prevents us from detecting MSI events. Second, we investigated the unstable loci in WES and RNA-seq to see if they had statistical significance in the other platform data. Some loci had KS test *P*-values less than 0.05 but not in FDR test. Although not significant in the FDR test, these loci still showed meaningful length variations in the other dataset, indicating MSI events. As a result, we quantified the number of unstable loci specifically present in WES and RNA-seq. WES-specific loci might not show changes in microsatellite length in RNA-seq because the genes in those regions are either not expressed or exhibit allele-specific expression [52]. In addition, previous studies have revealed that RNA-specific alterations appear in cancer-related pathways [53]. Similarly, we observed RNA-specific MSI events in cancer-related genes such as *MAZ* (MYC Associated Zinc finger gene), *INO80E* (DNA-repair related gene), and *RPL* genes (ribosome-related genes). These findings suggest that the RNA-specific loci, although few in number (four per sample on average), might have arisen due to RNA-specific alterations. By examining the discrepancy in MSI events between WES and RNA-seq, we observed that using loci in the CDS region for MSI detection in RNA-seq is suboptimal, consistent with our previous results. Thus, more accurate MSI detection markers are needed for RNA-seq. Our prior research has shown that loci in the 3′ UTR region provide better performance, indicating that, unlike in the DNA data, 3′ UTR loci are more suitable markers for MSI detection in RNA-seq. This highlights the importance of using diverse data types in MSI research. It should involve not only traditional genomic data but also transcriptomic, proteomic, epigenetic, and pathological imaging data. Integrating these diverse aspects for determining MSI status can provide more accurate diagnoses to patients and potentially enhance the efficacy of immunosuppressive treatments.

**Key Points**

- We present a novel method, MIRACLE, for measuring the lengths of microsatellites from RNA-seq to detect MSI.
- MIRACLE shows microsatellites in the 3′ UTR have the highest MSI detection predictive value.
- Integration of an RNA-based approach to MSI detection enables more accurate diagnoses.

## Supplementary Material

FigureS1_Final_version_bbae423

FigureS2_Final_version_bbae423

FigureS3_Final_version_bbae423

FigureS4_Final_version_bbae423

FigureS5_Final_version_bbae423

SuppleTable_Final_version_bbae423

## Data Availability

All data is contained within the manuscript and/or the supplementary materials. Code for MIRACLE is available at https://github.com/JinWookArgon/MIRACLE.
