# Supplementary figures and images for: Detecting microsatellite instability by length comparison of microsatellites in the 3′ untranslated region with RNA-seq
Source: Brief Bioinform. 2024 Aug 29;25(5):bbae423. doi: 10.1093/bib/bbae423 (PMC11361843; doi:10.1093/bib/bbae423)

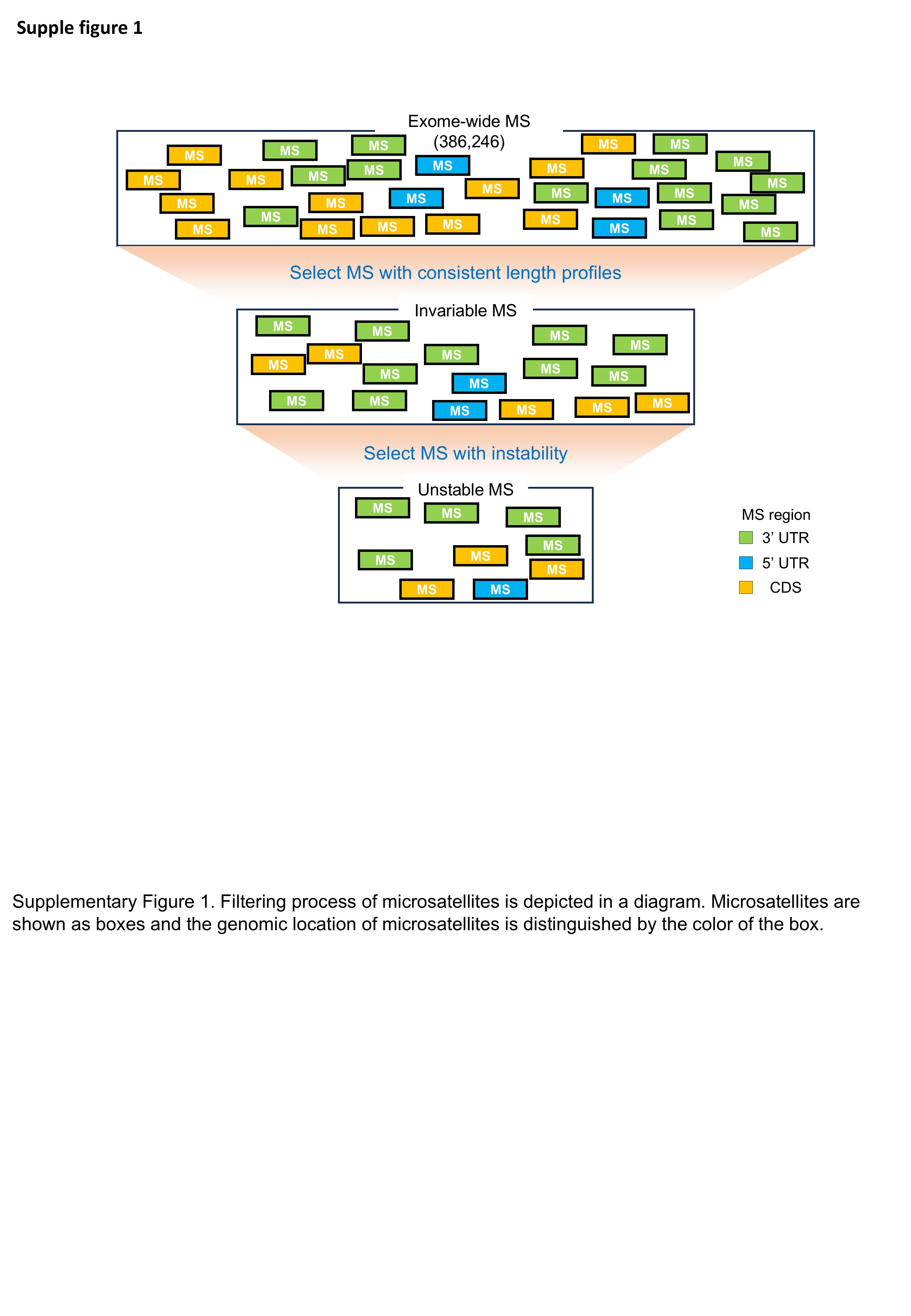

Supplement: FigureS1_Final_version_bbae423 [file figures1_final_version_bbae423.jpeg]

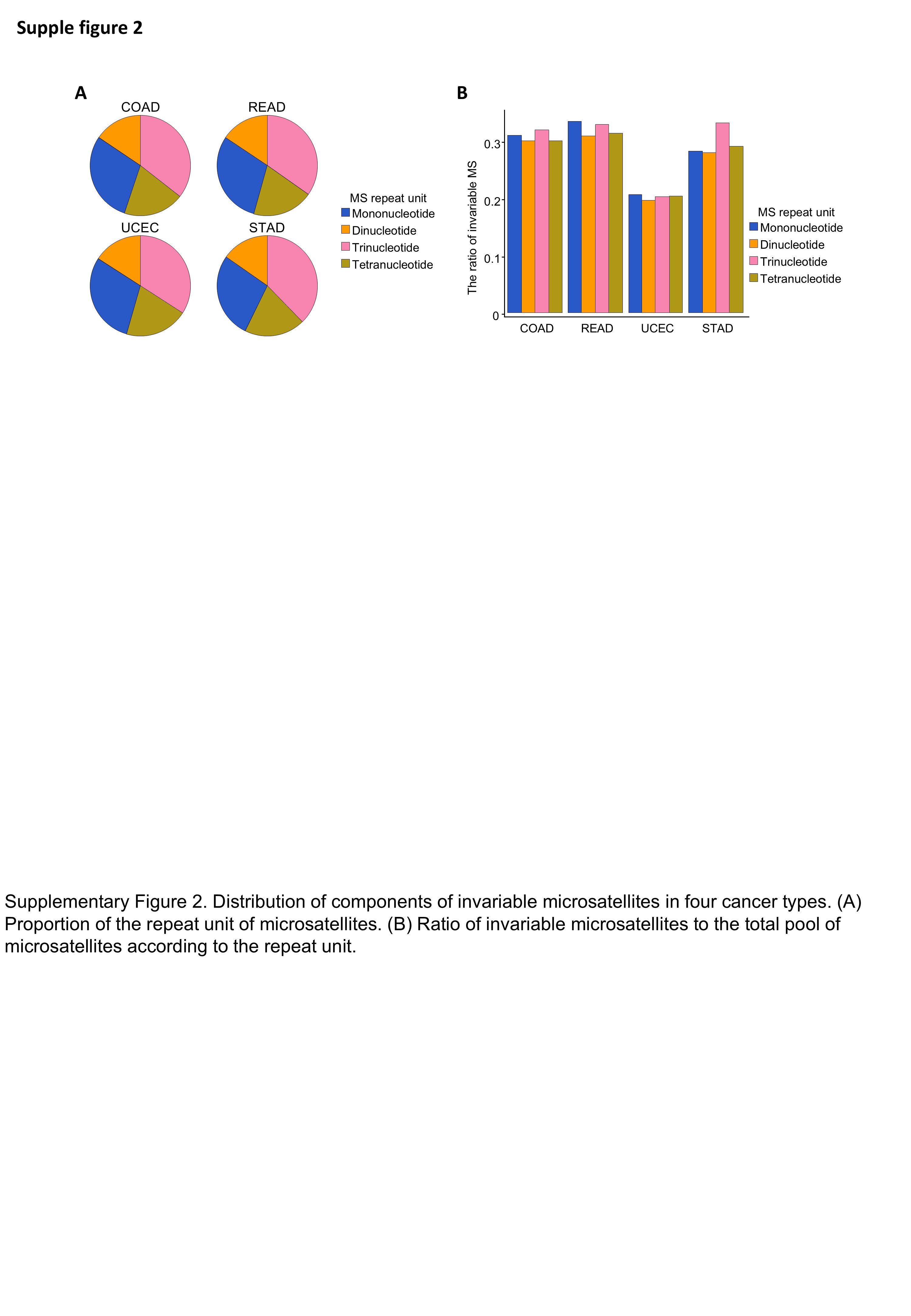

Supplement: FigureS2_Final_version_bbae423 [file figures2_final_version_bbae423.jpeg]

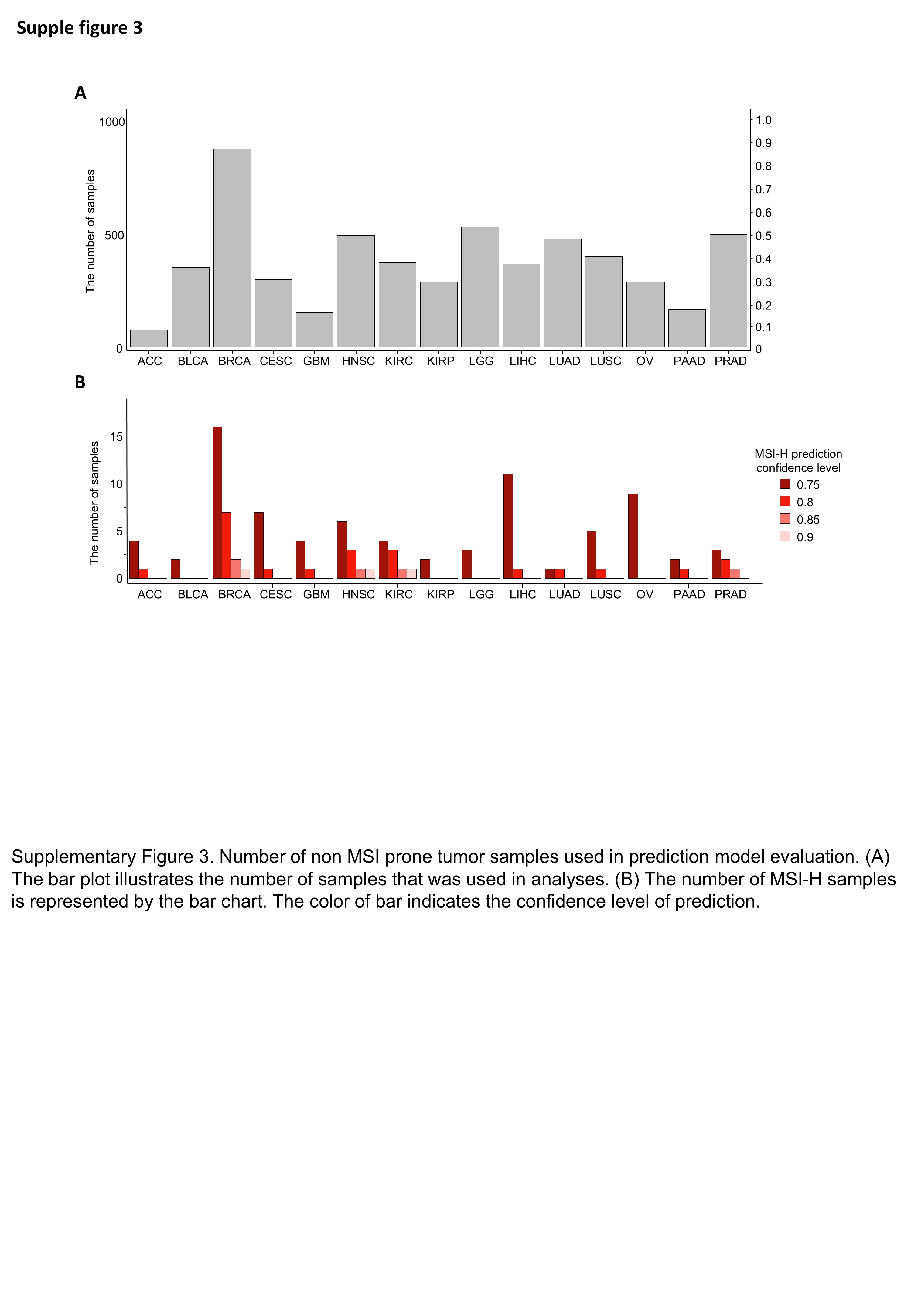

Supplement: FigureS3_Final_version_bbae423 [file figures3_final_version_bbae423.jpeg]

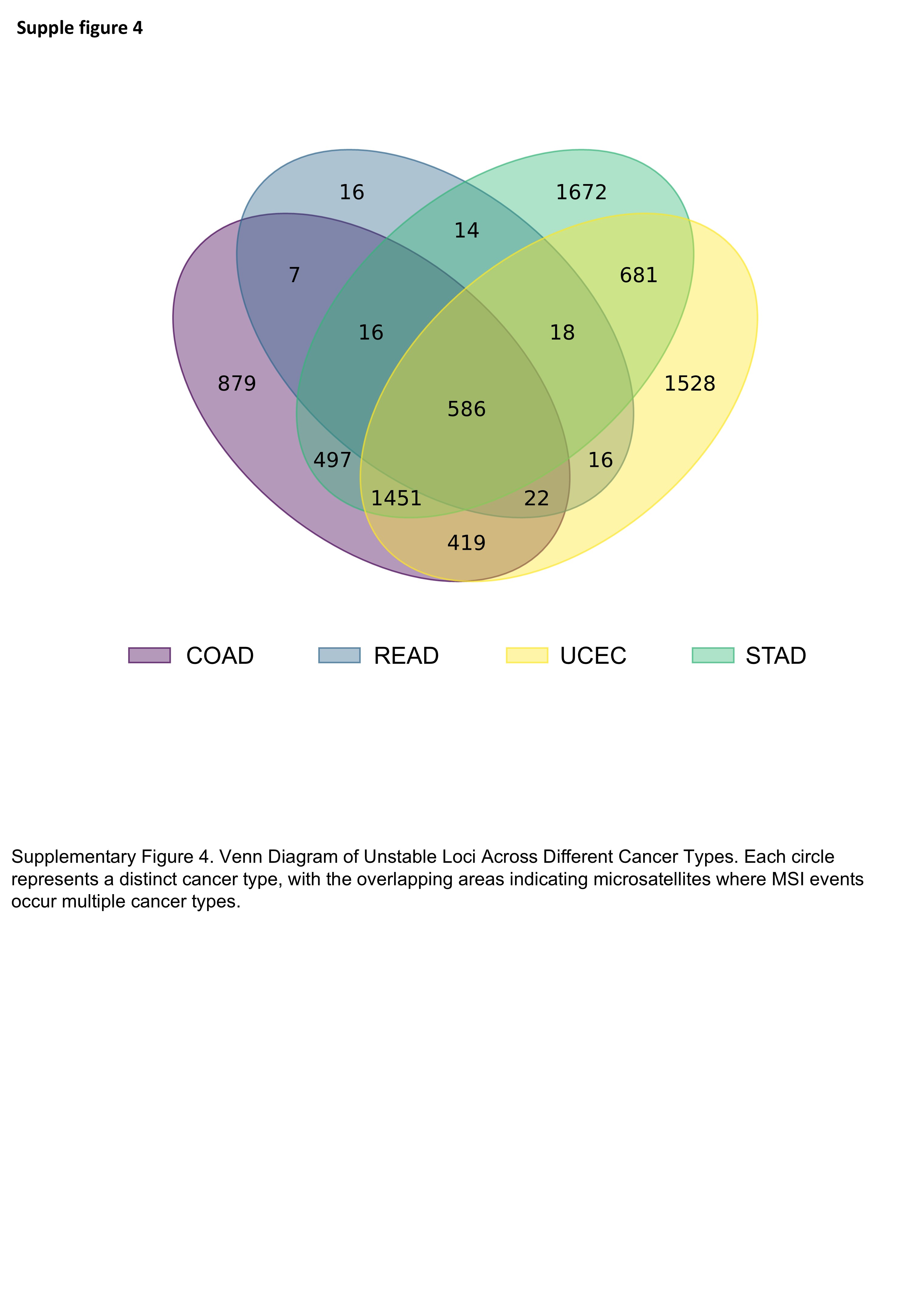

Supplement: FigureS4_Final_version_bbae423 [file figures4_final_version_bbae423.jpeg]

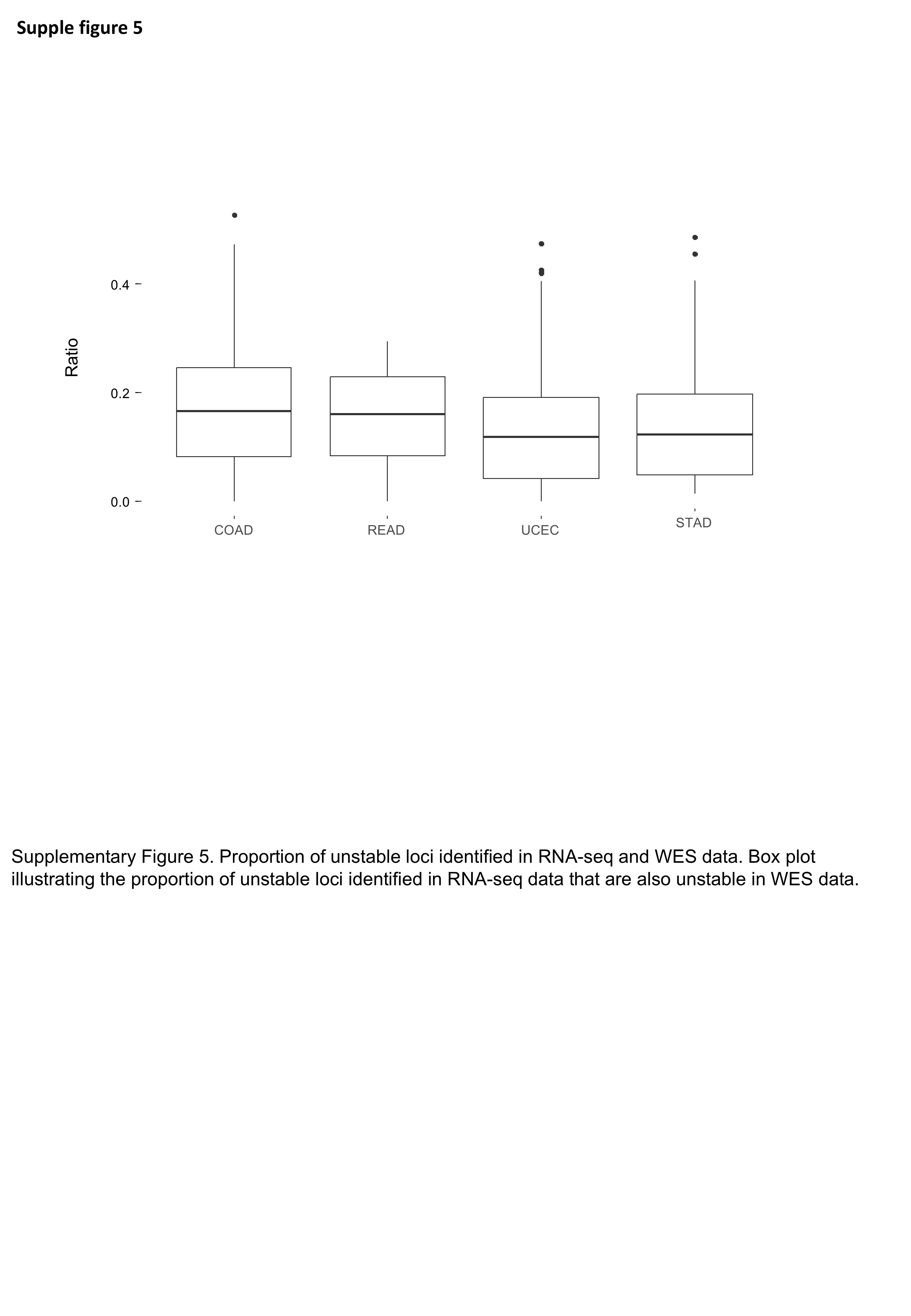

Supplement: FigureS5_Final_version_bbae423 [file figures5_final_version_bbae423.jpeg]
